# Risk and protective factors for substance use among Iranian university students: a national study

**DOI:** 10.1186/s13011-018-0181-2

**Published:** 2018-12-06

**Authors:** Farhad Taremian, Hamid Yaghubi, Hamid Pairavi, Seyed Ruhollah Hosseini, Masoud Zafar, Reza Moloodi

**Affiliations:** 1 Islamic Republic of, University of Social Welfare and Rehabilitation Science, Tehran, Iran; 2 Substance Abuse and Dependence Research Center, University of Social Welfare and Rehabilitation Sciences, Tehran, Iran; 3 Department of Clinical Psychology, University of Social Welfare and Rehabilitation Sciences, Tehran, Iran; 4 Counselling Center, University of Tehran, Tehran, Iran; 5 Shahed University Counseling Center, Tehran, Iran

**Keywords:** Drug abuse, Substance use, Risk factors, Prevention, University students, Protective factors

## Abstract

**Background:**

Substance use is a major mental health concern among university students. It may result in behavioral and academic problems, psychiatric disorders, and infectious diseases. Thus, this study investigated the risk and protective factors of substance use among Iranian university students.

**Methods:**

This was a cross-sectional study. A number of 7330 students were selected from 30 universities in Iran. The participants completed a researcher-designed questionnaire. It measured life time, previous year and previous month’s substance use, demographic characteristics, and a body of risk and protective factors including, religious beliefs, self-esteem, stress and psychological pressure, sensation seeking, attention seeking, anger and aggression, depression and anxiety, parents’ positive attitude towards substance use, lack of intimacy between family members, plus substance use, smoking cigarettes or hookah, alcohol consumption, and prescribed medications use by their family members, easy access to illegal drugs, peers’ positive attitude towards substance use, peers’ drug use, perceived prevalence of substance use among students, and negative attitude toward university. The data were analyzed using descriptive statistics and multivariate logistic regression analysis.

**Results:**

Participants’ anger and aggression, depression and anxiety, participants’ positive attitude towards substances, low level of religious beliefs, peers and family member’s substance use, and parent’s positive attitude towards substance significantly and strongly predicted using cigarette/hookah, alcohol, hard drugs, and prescribed medications. Having a negative attitude toward university significantly predicted using all types of substance (except for prescribed medications). Low self-esteem predicted using cigarette/hookah, and alcohol use. Perceived availability of illegal drugs predicted hard drugs and prescribed medications’ consumption. Finally, peers’ positive attitude toward drugs anticipated cigarette/hookah use.

**Conclusion:**

Prevention programs are most needed among Iranian students. They should be comprehensive in nature and focus on students’ psychoeducation about substances and their related negative consequences, plus promotion of students’ life skills, and integrate family- and peer-based preventive interventions.

## Background

Substance use is a primary mental health problem in the Iranian population. Studies have estimated that approximately 1.12 (2.1%) million Iranians aged 15–64 years old meet the Diagnostic and Statistical Manual for Mental Disorders-5 (DSM-5) criteria of substance use disorder [1]. Substance use is also a common problem among Iranian adolescents and students. Some studies have shown that 2% of Iranian high school students and 3.75% of Iranian university students [2] have ‌a lifetime problem of drug abuse [3].

The university years of life are accompanied by intense academic pressures, independence and limitation of parental supervision [4]. In this period exposure to illicit drugs increases [5]. Epidemiological studies indicate that substance use is a major public and social health problem among students [6–9]. For example, researches on university students of Tehran show a high life time prevalence of hookah (33.9%), cigarette (24%), alcohol (17%), opium (2.3%), and hashish/cannabis use (2.2%) [10–12]. These studies show that the commonness of cigarette/hookah, alcohol, hashish, and opium use are 13.2, 8.3, 0.8, and 0.58% among the university students in the past 12 months, respectively [11].

Among students, substance use has been associated with behavioral and academic problems, psychiatric disorders, and infectious diseases such as AIDS and hepatitis [12, 13]. Therefore, understanding risk and protective factors of substance use is a clinical and research necessity among students. This helps mental health practitioners and policy makers to develop effective preventive programs for substance abuse.

A number of longitudinal and cross-sectional studies have examined the risk and protective factors of student’s substance use. These studies consistently show that male gender [8, 14], internalizing and externalizing symptoms [15–17], positive attitude toward drugs [14, 18], and low level of religious beliefs [19, 20] are associated with an increased risk of substance abuse. In addition, interpersonal factors such as living in disadvantaged neighborhoods [21, 22], having a negative attitude toward university or school [14, 23], family members’ substance use history [21, 24], having parents who suffer from a psychopathology [21, 25], peer’s norms about substance use [26–28], are important risk factors for substance abuse among young adults.

However, to our knowledge, few studies have examined the risk and protective factors of substance abuse among young adults in Middle-Eastern cultures [7]. For example, Yi, Peltzer [7] conducted a multi-country cross-sectional study on 7923 students from nine southeast Asian countries. They found out that the low socioeconomic status, perceived poor health status and living away from parents are associated with an increased risk of substance use. Abu-Ras, Ahmed [29] found out that low religious activities and parents’ positive attitude toward alcohol use are major risk factors for drinking among Muslim students. A small study on Iranian university students showed that attitude towards substance use, sensation seeking, and impulsivity, friends’ positive attitude toward substance use, perceived accessibility, low level of family monitoring, and parents’ positive attitude toward substance use were the strongest predictors of substance use [30].

Considering scarce of knowledge about risk and protective factors of substance abuse among Iranian university students, this study investigated the risk and protective factors of substance use among Iranian university students.

## Methods

The population of this cross-sectional study were all students of undergraduate, postgraduate and doctoral programs of universities affiliated to the Ministry of Science, Research and Technology of Iran. This included 7700 university students. 370 participants had skipped more than 10% of the questionnaire items. Thus, the data of 7330 (3387 men and 3943 women) participants were analyzed.

### Sample size

The sample size of 7330 provided a high accuracy for estimating the past 12-month prevalence rate of opium use among Iranian student [11]. The 95% confidence interval width for this sample was 0.35 percentage points.

### Instruments

#### Drug use questionnaire

The drug use questionnaire (11) is a self-administered, Persian language instrument that was developed by the corresponding author of this study in 2008 [11]. The questionnaire consisted of six separate sections: 1) sociodemographic characteristics; 2) prevalence rate of substance use; 3) individual factors (including religious beliefs, self-esteem, attention seeking, stress and psychological pressure, sensation seeking, anger and aggression, depression and anxiety, individual’s attitude towards substance use; 4) family-related factors (including parents’ positive attitude towards substance use, lack of intimacy between family members, plus substance use, smoking cigarette or hookah, alcohol consumption, and prescribed medications use by the family members (; 5) social/environmental factors (including percived availability of illegal drugs, peers’ positive attitude towards substance use, peers’ drug use); and 6) university-related factors (including perceived prevalence of substance use among students and having a negative attitude towards university). The more detailed description is as follows:

1. **Sociodemographic characteristics:** This included sex, marital status, age, year of study, religion, father and mother’s educational status.
2. **Prevalence rate of substance use:** Life time, previous year, and previous month’s prevalence of substance use were measured by a questionnaire based on the American Drug and Alcohol Survey [31]. This scale assesses the substance use on a life time, annual, and past 30-day time frames. The validity and reliability of the Persian version of the scale has been established [10, 11].
3. **Individual factors:** This included anger and aggression (five items, for example “being beaten up by someone”), depression and anxiety (six items), an individual’s attitude towards substance, and religious beliefs (three items) that were adopted from the Prevention Planning Survey [32]. In our study, these subscales’ internal consistencies were from 0.84 (depression and anxiety) to 0.89 (anger and aggression) and in line with the previous studies [10, 11]. Self-esteem was measured using Single-Item Self-Esteem Scale (SISE) [33]. The SISE requires participants to answer the statement “I have high self-esteem” using a 5-point Likert-scale, ranging from “not at all true of me” to “very true of me.” Validity and reliability of the SISE was demonstrated [34, 35]. Stress and psychological pressure was assessed using 10 items concerning general psychological pressure (for example “I suffer a lot of stress” and “one of my concerns is finding the right job in the future”) and specific stresses associated with the campus (for example “behavior of the university officials/professors is not appropriate with the students”). Content validity of these items were confirmed by four psychologists. In our study, internal consistency of the stress and psychological pressure scale was 0.78. Sensation seeking subscale was adopted from the 12 items of the Zuckerman-Kuhlman Personality Questionnaire [36] (for example “I like “wild” uninhibited parties”, “I will try anything once”). The acceptable validity and reliability of this questionnaire has been shown in different communities [37, 38]. In our study, the internal consistency of the scale was 0.83. Finally, attention seeking was measured using three items (for example “I like getting attention from others”). The content validity of these items were again approved by four psychologists. The internal consistency of the scale was 0.74 and in line with previous studies [10, 11].
4. **Family-related factors:** The level of intimacy between family members was assessed with the family support and conflict subscale (10 items) of the prevention planning survey [32]. In our study, internal consistency of this subscale was 0.87. Also, parents’ positive attitude towards substance were measured using the family sanction subscale of prevention planning survey [32]. Finally, we asked participants to determine whether their father, mother, grandfather/mother, and siblings have used cigarette/hookah, alcohol, hard drugs (i.e., hashish, ecstasy, opium, crack, cocaine, (LSD), and methamphetamine), and prescribed medications during the previous 12 months.
5. **Social/environmental factors:** Perceived availability of illegal drugs was assessed by two items (for example “drugs/alcohol can be easily provided” and “drugs can be provided at a low cost”). Content validity of these items were confirmed by four psychologists. The students were asked to determine how much their peers would agree with smoking cigarette/hookah, hard drugs, or using alcohol and prescribed medications [32]. To assess peers’ drug use, students were asked to determine how many of their close friends smoked cigarette/hookah, or used alcohol, hard drugs, and prescribed medications during previous year.
6. **University-related factors:** Having a negative attitude towards university was measured with two items (“my expectations of the university have not been met” and “I regret getting into this university”). Finally, to assess the perceived prevalence of substance use we asked students the following question: “In your opinion, how much common is the use of these substances among students of your university?”. The options were cigarette/hookah, alcohol, hard drugs, and prescribed medications. The participants answered the question using a 4-point Likert scale (from 0 very low to 3 very high).

### Data collection procedure

A clinical psychologist from the counseling center of each of the 30 selected universities was recruited as assessor. They took part in an eight-hour workshop. The workshop’s aim was to train them about the purpose and procedure of the study, how to implement the questionnaire, and research ethics. Copied questionnaires, written consent, and written implementation guidelines were handed out to the assessors. They selected classes based on the year of the study of the students. They went to the classes and explained the purpose and procedure of the study to the students. Students who provided a written consent were asked to answer the questionnaire anonymously and put them in predetermined box.

### Statistical analysis

Data analyses were done using the statistical package for the social sciences (SPSS) software version 24. The logistic regression analysis was conducted to examine the effects of each independent variable on dependent variables, using each risk factor as an independent variable and each substance use as a dependent variable. All independent variables were simultaneously included in the model. We used adjusted odds ratios (aOR) and 95% confidence interval. The dependent variables were cigarette/hookah, alcohol, hard drugs, and prescribed medications use during the previous year. We considered students who had used substances in 12 months before the study as substance users. The independent variables were individual factors (including religious beliefs, self-esteem, attention seeking, stress and psychological pressure, sensation seeking, anger and aggression, depression and anxiety, individual’s attitude toward substance use), family-related factors (including parents’ attitude toward substance use, intimacy between the family members, substance use, smoking cigarette/hookah, alcohol consumption, and prescribed medications use by the family members, social/environmental factors (including the perceived availability of drugs, peers’ positive attitude toward substance use, and their drug use), and university-related factors (including perceived prevalence of substance use among students, and attitude toward university (for example, individual, family members, peers, and university factors). Descriptive statistics were used to describe sociodemographic characteristics of the participants as well as their life time, previous 12 months, and previous month’s prevalence rate of substance use.

#### Ethical considerations

Students answered the questionnaire anonymously. All participants signed a written consent. All assessors were payed for their cooperation. The research procedure was approved by the ethics review board of the Iran Drug Control Headquarter (IDCH).

## Results

### Sociodemographic characteristics

The mean age of men (*n* = 3387 (46.20%)) was 21.62 (*SD* = 2.53). For women (*n* = 3943 (53.80%)) this was 21.28 (*SD* = 2.36) years old. In terms of marital status, 87.12% of men and 80.77% of women were single. Most participants were Muslims (91.84% of men, and 92.54% of women) (Table 1).

**Table 1:** Demographic characteristics of students

	Male students (*n* = 3387 (46.20%))	Female students (n = 3943 (53.80%))	Total
*Marital status*			
Single	2951(87.12%)	3185 (80.77%)	6136 (83.71%)
Married	389 (11.48%)	703 (17.82%)	1092 (14.89%)
*Religion*			
Islam	3096 (91.840%)	3649 (92.54%)	6745 (92.01%)
Other religions	25 (0.73%)	16 (0.4%)	41 (0.55%)
Father education			
Illiterate	906 (26.74%)	908 (23.02%)	1814 (24.74%)
Completed high school	714 (21.08%)	1064 (26.98%)	1778 (24.25%)
Bachelor	866 (25.56%)	984 (24.95%)	1850 (25.23%)
Master degree or higher	259 (7.64%)	215 (5.45%)	474 (6.46%)
Mother Education			
Illiterate	1258 (37.14%)	1361 (34.51%)	2619 (35.72%)
Completed high school	730 (21.55%)	1071 (27.31%)	1801(24.57%)
Bachelor	579 (17.09%)	318(8.06%)	897 (12.23%)
Master degree or higher	110 (3.24%)	84 (2.13%)	194 (2.64%)

### Factors associated with alcohol use in the previous 12 months

There was a significant association between independent variables and alcohol use. The full model containing all predictors was statistically significant (*x*^*2*^(19, *N* = 3387) = 37.16, *p* < 0.001). The model explained 36.3% of the variance in alcohol use, and classified 92% of the cases. Family member’s alcohol use (aOR = 30.52, 95% CI = 12.27–75.86), family members’ drug use (aOR = 2.86, 95% CI = 0.98–8.31), peers’ substance abuse (aOR = 3.21, 95% CI = 2.50–4.12), and parents’ positive attitude toward substance use (aOR = 1.51, 95% CI = 1.31–1.72) had a statistically significant contribution to the model. In addition, students who had a positive attitude toward substance abuse (aOR = 2.59, 95% CI = 2.07–3.24), a negative attitude towards university (aOR = 1.54, 95% CI = 1.22–1.95), higher anger and aggression (aOR = 1.89, 99% CI:1.56–2.30), higher depression and anxiety (aOR = 1.36, 95% CI = 1.08–1.72), and lower self-esteem (aOR = 0.72, 95% CI: 0.58–0.89), and those with low level of religious beliefs (aOR = 1.97, 99% CI = 1.74–2.23) were more likely to have had used alcohol in the previous year (Table 2 and Table 3).

**Table 2:** Characteristics of user and non-user students according to predictor variables

Predictors	Substance Categories							
	Alcohol		Cigarette/Hookah		Hard drugs		Medications	
	Last year user	Non user	Last year user	Non user	Last year user	Non user	Last year user	Non user
	*N* = 577 N (%)	*N* = 6753 N (%)	*N* = 1459 N (%)	*N* = 5403 N (%)	*N* = 193 N (%)	*N* = 7137 N (%)	*N* = 807 N (%)	*N* = 6523 N (%)
Family members’ cigarette/hookah use	253 (43.8%)	1612 (23.9%)	600 (41.12%)	1114 (18.97%)	92 (47.7%)	1114 (15.60%)	297 (36.8%)	1114 (17.07%)
Family members’ alcohol use	132 (22.9%)	211 (3.1%)	169 (11.58%)	153 (2.6%)	53 (27.5%)	153 (2.14%)	70 (8.7%)	153 (2.34%)
Family members’ drug use	144 (25%)	197 (2.9%)	116 (7.59%)	148 (2.52%)	41 (21.2%)	148 (2.07%)	70 (8.7%)	148 (2.26%)
Family members’ prescribed medications use	144 (25%)	831 (12.3%)	298 (20.42)	586 (9.98%)	56 (29.01%)	586 (8.21%)	207 (25.7%)	586 (8.98%)
Peers’ drug use	245 (42.5%)	579 (8.6%)	399 (27.34%)	399 (6.79%)	142 (73.57%)	399 (5.59%)	163 (20.2%)	399 (6.11%)
Predictors	Substance Categories							
	Alcohol		Cigarette/Hookah		Hard drugs		Medications	
	Last year user Mean (SD)	Non user Mean (SD)	Last year user Mean (SD)	Non user Mean (SD)	Last year user Mean (SD)	Non user Mean (SD)	Last year user Mean (SD)	Non user Mean (SD)
Low level of religious beliefs	2.45 (0.92)	1.61(0.67)	2.08 (0.63)	1.64 (0.70)	2.46 (0.96)	1.62 (0.69)	1.91 (0.85)	1.64 (0.70)
self-esteem	2.08 (0.63)	2.97 (0.81)	2.08 (0.63)	2.94 (0.62)	2.06 (0.62)	2.31 (0.68)	2.08 (0.62)	2.12 (0.63)
Stress and Psychological pressure	2.94 (0.69)	2.91 (0.65)	2.96 (0.67)	2.92 (0.65)	2.89 (0.73)	2.92 (0.65)	3.04 (0.64)	2.92 (0.65)
Attention seeking	2.28 (0.60)	2.13 (0.60)	2.22 (0.57)	2.13 (0.58)	2.43 (0.66)	2.13 (0.58)	2.15 (0.56)	2.13 (0.58)
Sensation seeking	3.01 (0.59)	2.76 (0.58)	2.97 (0.56)	2.47 (0.51)	3.09 (0.61)	2.77 (0.57)	2.91 (0.59)	2.77 (0.57)
Depression and anxiety	2.70 (0.63)	2.45 (0.60)	2.78 (0.60)	2.48 (0.60)	2.76 (0.67)	2.48 (0.60)	2.74 (0.60)	2.48 (0.60)
Individual’s positive attitude towards substance abuse	1.95 (0.72)	1.30 (0.74)	1.76 (0.68)	1.33 (0.73)	2.37 (0.81)	1.33 (0.73)	1.60 (0.80)	1.33 (0.73)
Anger and aggression	2.09 (0.62)	1.66 (0.53)	1.99 (0.59)	1.70 (0.54)	2.29 (0.66)	1.70 (0.54)	1.92 (0.63)	1.70 (0.54)
Low level of intimacy between the family members	1.89 (0.66)	1.67 (0.57)	1.87 (0.65)	1.69 (0.57)	2.02 (0.79)	1.69 (0.57)	1.87 (0.64)	1.69 (0.57)
Parent’s positive attitude towards substance use	1.64 (0.76)	1.28 (0.73)	1.54 (0.70)	1.29 (0.72)	1.87 (0.99)	1.29 (0.72)	1.40 (0.75)	1.29 (0.72)
Peer’s positive attitude toward substance use	2.05 (0.75)	1.50 (0.79)	1.93 (0.72)	1.53 (0.78)	2.32 (0.85)	1.53 (0.78)	1.76 (0.82)	1.53 (0.78)
Perceived prevalence of substance use among students	2.06 (0.70)	1.89 (0.71)	1.97 (0.70)	1.90 (0.66)	2.31 (0.72)	1.9 (0.70)	2.06 (0.67)	1.90 (0.70)
Negative attitude toward the university	2.98 (0.50)	2.69 (0.52)	2.92 (0.51)	2.72 (0.52)	2.97 (0.52)	2.72 (0.52)	2.86 (0.51)	2.72 (0.52)
Perceived availability to illegal drugs	2.59 (0.85)	2.47 (0.88)	2.58 (0.82)	2.48 (0.87)	2.84 (0.82)	2.48 (0.87)	2.69 (0.81)	2.48 (0.87)

**Table 3:** Factors associated with substance use in the past 12 months among university students

Predictors	Substance Categories			
	Alcohol	Cigarette/Hookah	Hard drugs	Medications
	aOR (95% CI)	aOR (95% CI)	aOR (95% CI)	aOR (95% CI)
Low level of religious beliefs	1.976^**^ (1.74–2.23)	1.39^*^ (1.22–1.51)	1.24^*^ (1.01–1.51)	1.02 (0.97–1.2)
Low self-esteem	0.723^*^ (0.58–0.89)	0.64^*^ (0.54–0.76)	1.00 (0.71–1.43)	0.79 (0.68–0.94)
Stress and Psychological pressure	0.76 (0.62–0.91)	0.79 (0.67–0.92)	1.65^*^(2.63–4.96)	0.17 (0.87–1.18)
Attention seeking	0.89 (0.72–1.11)	0.92 (0.79–1.06)	1.02 (0.72–1.44)	0.78 (0.66–0.92)
Sensation seeking	0.89 (1.02–1.52)	1.30^*^ (1.14–1.43)	1.49^*^ (1.08–2.05)	1.23^*^ (0.88–1.53)
Depression and anxiety	1.36^**^ (1.08–1.72)	1.30^*^ (CI = 1.11–1.52)	0.92 (0.63–1.35)	1.68^*8^ (1.41–2.02)
Individual’s positive attitude towards substance abuse	2.59^**^ (2.07–3.24)	3.08^** (^2.63–4.96)	3.62^**^ (2.63–4.96)	1.57^* (^1.29–1.93)
Anger and aggression	1.89^**^ (1.56–2.30)	1.76^** (^1.53–2.03)	1.63^*^ (1.20–2.22)	1.19^*^ (1.02–1.40)
Low level of intimacy between the family members	0.96 (0.8–1.15)	1.12 (1.03–1.42)	0.99 (0.85–1.51)	1.03 (0.95–1.25)
Family members’ cigarette/hookah use	0.48 (0.34–0.63)	7.52^*^ (4.97–11.36)	4.33^**^ (1.73–10.86)	2.34^**^ (1.45–3.77)
Family members’ alcohol use	30.52^**^ (12.27–75.86)	2.24^*^ (1.02–4.88)	7.05^**^ (2.10–23.57)	0.06 (0.00–0.12)
Family members’ drug use	2.86^*^ (0.98–8.31)	2.71^**^ (CI = 1.16–6.30)	12.07^**^ (2.88–50.72)	5.03^**^ (2.12–11.95)
Family members’ prescribed medications use	1.09 (0.92–1.22)	1.70^* (^= 1.28–2.83)	0.66 (0.41–0.90)	2.83^*^ (1.69–4.75)
Parent’s positive attitude towards substance use	1.51^**^ (1.31–1.72)	1.34^**^ (1.13–1.57)	0.65 (0.50–0.85)	1.73^*^ (1.41–2.05)
Peer’s positive attitude toward substance use	1.08 (0.9–1.24)	1.23^*^ (1.08–1.40)	0.76 (0.56–1.01)	0.93 (0.79–1.07)
Peers’ drug use	3.21^**^ (2.50–4.12)	2.77** (2.22–3.46)	3.15^**^ (2.29–4.31)	1.26^*^ (0.99–1.60)
Perceived prevalence of substance use among students	1.07 (0.89–1.29)	0.95 (0.81–1.07)	3.15^**^ (2.29–4.31)	1.20^*^ (1.06–1.35)
Negative attitude toward the university	1.54** (1.22–1.95)	1.4* (1.17–1.68)	1.56^*^ (1.08–2.31)	1.01 (0.85–1.20)
Perceived availability to illegal drugs	0.98 (0.85–1.11)	1.05 (0.92–1.14)	1.28^*^ (1.03–1.60)	1.18^*^ (1.06–1.3)

*aOR* Adjusted Odds Ratio; * *p* < 0.05; ** *p* < 0.01

### Factors associated with cigarette/hookah use in the previous 12 months

Multivariate logistic regression analysis for exploring factors associated with smoking cigarette/hookah also showed a significant contribution of independent variables (*x*^*2*^ = (19, *N* = 7330) = 62.752, *p* < 0.001) (Table 2). The model explained 29% of the variance in cigarette/hookah use and classified 88.5% of cases correctly. Smoking cigarette or hookah by the family members (aOR = 7.52, 95% CI = 4.97–11.36), family members’ drug use (aOR = 2.71,95% CI = 1.16–6.30), alcohol consumption (aOR = 2.24, 95% CI = 1.02–4.88), and prescribed medications use (aOR = 1.70, 95% CI = 1.28–2.83), peers’ drug use (aOR = 2.77, 95% CI = 2.22–3.46), parents’ (aOR = 1.34, 95% CI = 1.13–1.57) and peers’ (aOR = 1.23, 95% CI = 1.08–1.40) positive attitude toward substance significantly predicted smoking cigarette/hookah use among the studied students. Also, there was more probability that students who expressed positive attitude toward substance use (aOR = 3.08, 95% CI = 2.53–3.75), negative attitude toward university (aOR = 1.4, 95% CI = 1.20–1.62), higher anger and aggression (aOR = 1.76, 95% CI = 1.53–2.03), higher sensation seeking (aOR = 1.30, 95% CI = 1.14–1.43), higher depression and anxiety (aOR = 1.36, 95% CI = 1.11–1.52) had more likely smoked cigarette/hookah during the previous 12 months (Table 2 and Table 3).

### Factors associated with hard drugs use in the previous 12 months

Results of the logistic regression showed that the independent variables used in the model for predicting hard drugs (hashish, ecstasy, opium, crack, cocaine, LSD, and methamphetamine) use had a significant influence (*x*^*2*^ (19, *N* = 7330) = 15.31, *p* < 0.01) (Table 2). The model accounted for 36% of variance of hard drugs use. Students who had a positive attitude towards substance use (aOR = 3.62, 95% CI:2.63–4.96), negative attitude toward university (aOR = 1.56, 95% CI:1.08–2.31), higher anger and aggression (OR = 1.63, 95% CI:1.20–2.22), higher stress and psychological pressure (aOR = 3.62, 95% CI:2.63–4.96), higher sensation seeking (aOR = 1.49, 95% CI:1.08–2.05), and lower obedience to religious rules (aOR = 1.24, 95% CI:1.01–1.51) had more significantly used hard drugs in the previous year. In addition, students who reported that their family members use hard drugs (aOR = 12.07, 95% CI:2.88–50.72), alcohol (aOR = 7.05, 95% CI:2.10–23.57), and cigarettes/hookah (aOR = 4.33, 95% CI:1.73–10.86) were more inclined to use hard drugs. Finally, peers’ drug use (aOR = 3.15, 95% CI:2.29–4.31), perceived prevalence of substance use in university (aOR = 3.15, 95% CI: 2.29–4.31), and accessibility of substance (aOR = 1.28, 95% CI: 1.03–1.60) were significantly associated with hard drug use (Table 2 and Table 3).

### Factors associated with prescribed medications use in the previous 12 months

The results of logistic regression showed that independent variables entered in the model significantly predict the prescribed medications use (*x*^*2*^ = (19, *N* = 7330) = 3.640, *p* < 0.01) (Table 2). The model explained 10% of the variance of the prescribed medications use. Students who had a positive attitude toward drugs (aOR = 1.57, 95% CI: 1.29–1.93), higher sensation seeking (aOR = 1.23, 95% CI: 0.88–1.53), higher anger and aggression (aOR = 1.19, 95% CI:1.02–1.40), and higher depression and anxiety (aOR = 1.68, 95% CI: 1.41–2.02) had more likely abused prescribed medications during the previous year. Also students who reported that their family members use hard drugs (aOR = 5.03, 95% CI:2.12–11.95), prescribed medications (aOR = 2.83, 95% CI:1.69–4.75), and cigarette or hookah (aOR = 2.34, 95% CI:1.45–3.77) were more likely to have abused prescribed medications during the previous 12 months. Finally, peers’ drug use (aOR = 1.26, 95% CI:0.99–1.60), and parents’ positive attitude toward drugs (aOR = 1.73, 95% CI: 1.41–2.05) was significantly associated with students prescribed medications use (Table 2 and Table 3).

## Discussion

To our knowledge, this is the first study that investigated risk and protective factors of substance use among Iranian university students. Identification of risk and protective factors of substance use is the first step in planning for prevention programs.

Our results showed that individual factors such as anger and aggression, and depression and anxiety significantly predict all types of substance use during the previous 12 months. These findings might be explained by the two factor avoidance theory [39]. According to this theory, negative affects serve as conditioned stimuli (CS) that elicit conditioned drug responses (CRs). These conditioned responses motivate an individual to engage in substance use to decrease his/her negative affects. Also, an individual’s positive attitude towards substance significantly anticipate all types of substance use during the previous year. According to the social learning model [40, 41], positive outcome expectations and lack of effective coping skills have an important role in maintaining substance use. Additionally, low level of religious beliefs and higher sensation seeking are associated with an increased use of cigarette/hookah and hard drugs. Several studies have found out that religious beliefs have an important role in preventing risky behaviors [14, 16–20]. Sensation seeking significantly predicted cigarette/hookah and hard drugs use. Students with low self-esteem are at an increased risk of alcohol and cigarette/hookah use. These findings imply that prevention programs should focus on interventions which include increasing self-esteem, life skill based drug education, and cognitive and emotional skills (for example stress management, anger management, and communication skills) [13, 42].

Among family-related factors, family member’s alcohol and hard drugs use, as well as parents’ positive attitude towards substance use are significantly and strongly associated with all types of substance use among students. Also, family members’ cigarette/hookah and prescribed medications use predicts using all types of substances among students (except for alcohol use). These results are consistent with previous findings [21, 24]. The results emphasize the role of family member’s substance use and their positive attitudes on substance use among university students and imply that preventive interventions should focus on family factors [13, 43]. However, we did not find a significant relationship between the extent of intimacy between family members and substance use. Further longitudinal researches need to investigate this issue.

Consistent with previous studies [26–28], our findings show that peers’ substance use strongly predicts all types of substance use among university students. Also, peers’ positive attitude toward drugs predicts prescribed medications use and smoking cigarette/hookah. Monahan, Steinberg, and Cauffman [44] discussed that adolescents are highly vulnerable to peer influence. These results are also consistent with theoretical models of peer influence (for example implying an active, explicit form of peer influence) [45]. Therefore, prevention programs should focus on reducing negative peer influence by training people regarding refusal and resistance skills [13, 42, 45].

Our findings show that having a negative attitude towards university increases the risk of alcohol, cigarettes/hookah, and hard drugs use among students. In other words, students who feel they do not belong to the university community are at greater risk for substance use. These results are similar to previous findings [46, 47]. It has been shown that individuals who feel attached to and interact with others in community, experience better physical and mental health [48]. This social attachment improves health via feelings of mutual respect and self-esteem, and exposure to healthy behaviors such as physical activity [49]. In addition, easy access to illegal drugs significantly predicts use of hard drugs and prescribed medications. These findings replicate the results of previous studies in other cultures [50, 51].

Generally, the results of our study can be explained by the theories of planned behavior [52, 53] and social learning. Ajzen [52, 53] argued that an individual’s intention is the critical component of behavior. According to this theory, three factors determine an individual’s behaviors: attitudes (for example an individual’s positive attitude towards drugs), normative expectations of important others (for example family members’ substance use, peers’ substance use, and parents and peers’ positive attitude towards drugs), and perceived behavioral control (for example self-efficacy expectations or perceptions of behavioral control). Moreover, social learning theory asserts that behavior is learned through relationships with social groups that provide role models, support, and reinforce the behavior [40, 41]. Thus, an adolescent who lives in a family or social group that they engage in substance use behaviors probably assumes that the family or social group expect him/her to do so without blaming him/her [54].

Although our study had a large-scaled national sample of students, the cross-sectional nature of the study hinders to deduce casual relationships between the variables. Therefore, longitudinal researches are needed to explore the causal role of risk and protective factors of substance use among Iranian university students.

## Conclusion

Our results indicated that individuals’ anger and aggression, depression and anxiety, positive attitude toward substances, low level of religious beliefs, peers and family member’s substance use, and parent’s positive attitude toward substance can significantly and strongly predict cigarette/hookah, alcohol, hard drugs, and prescribed medications use. In addition, low self-esteem, peers’ positive attitude toward drugs, negative attitude toward university, and easy access to illegal drugs anticipated using some categories of substances. Therefore, in an individual level, prevention programs should provide motivational intervention and normative feedback intervention to the students [55], as empirical evidences suggest motivational interventions result in more favorable drinking outcomes than students who receive direct education about drugs [56]. Campus interventions can also focus on promotion of students’ life skills (for example stress management, problem solving, and assertive skills) [57] and their social networks. In addition, university prevention programs should implement strategies to reduce availability to cigarette/hookah, alcohol, and other drugs and reduce community’s encouraging norms to substance use [58]. Finally, university based prevention interventions could incorporate family based preventive interventions [59].

## Abbreviations

IDCH: Iran Drug Control Headquarter
aOR: adjusted Odds Ratio
SD: Standard deviation
